# Supplementary material for: CryoFSL: An Annotation-Efficient, Few-Shot Learning Framework for Robust Protein Particle Picking in Cryo-EM Micrographs
Source: bioRxiv. 2025 Sep 21:2025.09.19.677446. Preprint. [Version 1] doi: 10.1101/2025.09.19.677446 (PMC12458156; doi:10.1101/2025.09.19.677446)
Supplement: Supplement 1 [file media-1.pdf]

CryoFSL: An Annotation-Efficient, Few-Shot Learning Framework for Robust Protein Particle Picking in Cryo-EM Micrographs

Biplab Poudel[1,2], Rajan Gyawali[1], Ashwin Dhakal[1], Jianlin Cheng[1*], Dong Xu[1,2*]

[1]Department of Electrical Engineering and Computer Science, University of Missouri, Columbia, MO 65211, USA
[2]Bond Life Sciences Center, University of Missouri, Columbia, MO 65211, USA
[*]Corresponding authors: Jianlin Cheng (jianlin.cheng@missouri.edu); Dong Xu (xudong@missouri.edu)

Supplementary Material

Supplementary Table S1: An overview of EMPIAR IDs from CryoPPP used for evaluation of CryoFSL (* represents theoretical weight of the proteins)

SN	EMPIAR ID	Type of Protein	Image Size	Total Structure Weight (kDa)	Number of Images
1	10028	Ribosome (80S)	(4096, 4096)	2135.89	300
2	10081	Transport Protein	(3710, 3838)	298.57	300
3	10345	Signaling Protein	(3838, 3710)	244.68	295
4	11056	Transport Protein	(5760, 4092)	88.94	305
5	10093	Membrane Protein	(3838, 3710)	779.4	295
6	10017	β-galactosidase	(4096, 4096)	450*	84
				**Total**	**1,879**

Supplementary Table S2: Efficiency of manually annotated particles on 3D resolution for sample proteins using template-based methods and CryoFSL.

EMPIAR ID	% of Manual Annotation	Number of Particles	EMAN2	RELION	Scipion	CryoFSL
10017	10%	170	6.14	5.62	6.15	5.10
	20%	341	6.09	5.92	6.19	5.17
	30%	513	6.06	5.76	6.08	5.32
	40%	684	6.08	5.70	6.04	5.01
	50%	857	5.92	5.65	5.96	5.14
	100%	1715	5.61	5.58	5.89	4.99
10081	10%	59	8.70	8.53	7.92	6.53
	20%	120	7.87	8.21	7.64	6.58
	30%	180	7.98	8.39	7.71	6.45
	40%	241	7.40	8.30	7.54	6.33
	50%	303	7.37	8.31	7.43	6.32
	100%	607	7.26	8.00	7.36	6.16

For each protein sample, we investigated the effect of varying the proportion of manually annotated particles on the achievable 3D resolution. The "% of manual annotation" column does not represent the fraction of total training data used; rather, it indicates the fraction of annotated particles selected from each micrograph within the CryoPPP dataset. Specifically, for every micrograph, we subsampled 10%, 20%, 30%, 40%, 50%, and a complete set of high-confidence annotated particles, and then combined these across five micrographs per protein to construct the training or template generation sets. For example, in the case of EMPIAR-10017, selecting 10% of particles corresponds to a total of 170 particles aggregated from the five micrographs (not 170 particles per micrograph). This subsampling strategy allowed us to examine how the resolution scales with increasingly larger sets of particles while keeping the number of micrographs fixed. The resulting particle counts are reported in the "Number of particles" column, which served either as the input for training CryoFSL or as the reference templates for conventional template-based methods. Doing so ensured a fair comparison across methods under varying annotation efficiencies.

Supplementary Table S3: Quantitative performance of particle picking across representative micrographs from EMPIAR-10345. Each row reports the expert-curated ground truth (GT) particle count, alongside predicted particle numbers (Pred.), precision (Prec), and recall (Recall) for different methods. The table illustrates the variability in prediction behaviors at the micrograph level, complementing the global statistical analyses presented in the main manuscript.

Micrograph name		CrYOLO			Topaz			Scipion			RELION			EMAN2			CryoFSL		
	GT	Pred.	Prec	Recall	Pred.	Prec	Recall	Pred.	Prec	Recall	Pred.	Prec	Recall	Pred.	Prec	Recall	Pred.	Prec	Recall
18jam15a_0176_ali_DW	39	26	0.2017	0.1327	83	0.01437	0.02531	653	0.06039	0.8694	709	0.0654	0.9153	104	0.12346	0.314797	134	0.2634	0.9728
18jam15a_0185_ali_DW	16	14	0.0492	0.0408	363	0.00613	0.09916	646	0.02612	0.8959	185	0.07648	0.79825	147	0.027868	0.238854	95	0.1682	0.8361
18jam15a_0205_ali_DW	29	12	0.11761	0.04715	23	0.00961	0.00686	683	0.04242	0.84321	756	0.046	0.895	156	0.124418	0.640653	112	0.2459	0.9551
18jam15a_0225_ali_DW	29	15	0.10899	0.05653	52	0.05997	0.10276	669	0.04652	0.9141	958	0.04258	0.9533	432	0.061367	0.82755	118	0.2401	0.9949
18jam15a_0230_ali_DW	19	13	0.00013	0.01	7	0.04799	0.01768	681	0.02967	0.8943	567	0.03664	0.89792	335	0.041677	0.661564	112	0.1436	0.9744
18jam15a_0231_ali_DW	24	14	0.05117	0.03006	5	0.01957	0.00411	641	0.0358	0.8329	532	0.05091	0.92507	421	0.046974	0.757085	126	0.179	0.9335
18jam15a_0249_ali_DW	17	17	0.0819	0.0819	133	0.02642	0.1841	692	0.0248	0.8422	281	0.046	0.6887	274	0.015231	0.222002	95	0.1631	0.9791
18jam15a_0264_ali_DW	22	15	0.03221	0.02196	199	0.00331	0.02218	658	0.0331	0.83659	650	0.0416	0.9668	223	0.05107	0.483323	106	0.19309	0.9462
18jam15a_0281_ali_DW	23	16	0.04904	0.03335	15	0.0013	0.0021	674	0.0344	0.8545	753	0.03706	0.9046	267	0.064406	0.689721	123	0.1665	0.989
18jam15a_0282_ali_DW	20	9	0.11127	0.05032	11	0.031	0.1634	663	0.02885	0.82097	622	0.03537	0.8886	436	0.031198	0.624682	97	0.1865	0.9961
18jam15a_0293_ali_DW	33	20	0.12288	0.0751	14	0.0051	0.1305	667	0.0494	0.8598	835	0.0525	0.9666	447	0.057713	0.717525	162	0.1784	0.9571
18jam15a_0295_ali_DW	39	9	0.13512	0.03074	18	0.0196	0.0054	660	0.0617	0.8853	772	0.06466	0.9504	142	0.1226	0.416327	146	0.1959	0.9793
18jam15a_0309_ali_DW	36	22	0.16515	0.10139	24	0.01437	0.0091	645	0.0563	0.868	698	0.0622	0.9245	278	0.108209	0.789765	146	0.18821	0.9814
18jam15a_0313_ali_DW	12	11	0.08121	0.07502	283	0.01362	0.2383	680	0.017	0.828	342	0.0344	0.8824	281	0.019406	0.412907	46	0.19182	0.97442

To further illustrate the robustness of particle picking methods, we examined 14 representative micrographs from EMPIAR-10345, reporting ground truth (GT) particle count, predicted counts, precision, and recall. Unlike aggregate evaluations that average over many micrographs, this micrograph-level analysis further highlights method-specific behaviors under varying particle densities and image conditions. An important observation from this table is that methods differ not only in aggregate performance but also in consistency across micrographs. For instance, CryoFSL exhibits a narrow-predicted particle range, remaining stable even when ground truth particle counts vary. In contrast, methods like Topaz, EMAN2, and RELION show large fluctuations in predicted counts relative to GT, indicating sensitivity to micrograph-specific factors such as noise patterns or particle clustering. For template-based methods, this manifests as uniformly inflated counts across nearly all micrographs, while Topaz alternates between extreme under-picking (as few as 5, 7) and over-picking (200, 300), reflecting instability in thresholding or learned representation. CrYOLO tends to scale its detections proportionally with particle density but systematically under-recovers absolute particle numbers in low SNR regions, which explains its low recall despite reasonable proportionality with GT. These divergent failure modes have practical consequences: aggressive over-picking from template methods introduces false positives that can degrade downstream alignment and classification, while under-picking methods risk losing true particle diversity needed for high-resolution reconstructions.

Supplementary Table S4: Precision comparison of particle picking methods across EMPIAR datasets using Wilcoxon tests. The table reports median precision from CryoFSL (focal) and competing methods, along with p-values, effect sizes, and FDR-adjusted p-values.

Dataset	Competitor	Test	n	Median_Focal	Median_Comp	p-value	Effect Size	Adj. p-value
10017	CrYOLO	Wilcoxon	79	0.80486	0.79467	0.55103	0.18987	0.55103
	Topaz	Wilcoxon	79	0.80486	0.78428	0.044065	0.24051	0.047212
	Scipion	Wilcoxon	79	0.80486	0.52516	1.502e-14	0.94937	1.7331e-14
	RELION	Wilcoxon	79	0.80486	0.37835	1.289e-14	0.94937	1.6113e-14
	EMAN2	Wilcoxon	79	0.80486	0.62842	1.4457e-14	0.92405	1.7331e-14
10028	CrYOLO	Wilcoxon	293	0.68852	0.77012	8.4968e-50	-1.00000	3.1863e-49
	Topaz	Wilcoxon	293	0.68852	0.68914	0.054719	0.00341	0.056606
	Scipion	Wilcoxon	293	0.68852	0.45003	8.4968e-50	1.00000	3.1863e-49
	RELION	Wilcoxon	293	0.68852	0.50424	8.4968e-50	1.00000	3.1863e-49
	EMAN2	Wilcoxon	293	0.68852	0.44749	8.4968e-50	1.00000	3.1863e-49
10081	CrYOLO	Wilcoxon	295	0.68893	0.67734	3.3951e-08	0.32881	3.7724e-08
	Topaz	Wilcoxon	295	0.68893	0.62652	9.35e-39	0.70847	1.3357e-38
	Scipion	Wilcoxon	295	0.68893	0.37692	4.0001e-50	1.00000	3.1863e-49
	RELION	Wilcoxon	295	0.68893	0.37310	4.0825e-50	0.99322	3.1863e-49
	EMAN2	Wilcoxon	295	0.68893	0.35851	4.2961e-50	0.98644	3.1863e-49
10093	CrYOLO	Wilcoxon	285	0.46324	0.54995	1.8433e-48	-0.97895	3.6866e-48
	Topaz	Wilcoxon	285	0.46324	0.53729	5.0293e-41	-0.75439	7.9409e-41
	Scipion	Wilcoxon	285	0.46324	0.33493	1.7302e-48	1.00000	3.6866e-48
	RELION	Wilcoxon	285	0.46324	0.38330	4.8601e-39	0.81053	7.2902e-39
	EMAN2	Wilcoxon	285	0.46324	0.39086	5.6256e-48	0.97193	1.0548e-47
10345	CrYOLO	Wilcoxon	291	0.39076	0.13676	2.2891e-49	0.97938	5.2825e-49
	Topaz	Wilcoxon	291	0.39076	0.03683	1.8049e-49	1.00000	4.5122e-49
	Scipion	Wilcoxon	291	0.39076	0.07499	1.8049e-49	1.00000	4.5122e-49
	RELION	Wilcoxon	291	0.39076	0.10302	1.8049e-49	1.00000	4.5122e-49
	EMAN2	Wilcoxon	291	0.39076	0.12292	1.8049e-49	1.00000	4.5122e-49
11056	CrYOLO	Wilcoxon	299	0.58650	0.68749	8.8662e-51	-1.00000	2.6599e-49
	Topaz	Wilcoxon	299	0.58650	0.63838	1.2757e-45	-0.83946	2.1261e-45
	Scipion	Wilcoxon	299	0.58650	0.49802	1.1612e-17	0.74582	1.5834e-17
	RELION	Wilcoxon	299	0.58650	0.38330	1.3175e-47	0.75251	2.3250e-47
	EMAN2	Wilcoxon	299	0.58650	0.51832	1.2994e-15	0.58528	1.6948e-15

Supplementary Table S5: Recall comparison of particle picking methods across EMPIAR datasets using Wilcoxon tests. The table reports median precision from CryoFSL (focal) and competing methods, along with p-values, effect sizes, and FDR-adjusted p-values.

Dataset	Competitor	Test	n	Median_Focal	Median_Comp	p-value	Effect Size	Adj. p-value
10017	CrYOLO	Wilcoxon	79	0.9557	0.38146	1.1491E-14	1	1.8852E-14
	Topaz	Wilcoxon	79	0.9557	0.71639	1.3915E-14	0.94937	2.0873E-14
	Scipion	Wilcoxon	79	0.9557	0.84421	3.1929E-12	0.92405	4.5613E-12
	RELION	Wilcoxon	79	0.9557	0.74916	3.4224E-12	0.92405	4.6669E-12
	EMAN2	Wilcoxon	79	0.9557	0.48612	1.1940E-14	0.97468	1.8852E-14
10028	CrYOLO	Wilcoxon	293	0.9187	0.80612	8.4968E-50	1	8.4968E-49
	Topaz	Wilcoxon	293	0.9187	0.8817	8.3895E-39	0.73379	2.0974E-38
	Scipion	Wilcoxon	293	0.9187	0.93211	3.5825E-01	-0.06485	3.7061E-01
	RELION	Wilcoxon	293	0.9187	0.92361	6.4858E-01	-0.04437	6.4858E-01
	EMAN2	Wilcoxon	293	0.9187	0.87667	5.4823E-36	0.75427	1.2652E-35
10081	CrYOLO	Wilcoxon	295	0.8848	0.69059	4.3845E-50	0.98644	6.5768E-49
	Topaz	Wilcoxon	295	0.8848	0.87729	1.9602E-01	0.11186	2.1002E-01
	Scipion	Wilcoxon	295	0.8848	0.81501	5.5174E-33	0.66102	1.1823E-32
	RELION	Wilcoxon	295	0.8848	0.81982	4.9136E-18	0.45085	9.2131E-18
	EMAN2	Wilcoxon	295	0.8848	0.8339	5.6711E-18	0.45763	1.0008E-17
10093	CrYOLO	Wilcoxon	285	0.6875	0.48477	5.3573E-48	0.98947	2.2960E-47
	Topaz	Wilcoxon	285	0.6875	0.52658	8.7489E-41	0.75789	2.3861E-40
	Scipion	Wilcoxon	285	0.6875	0.61673	5.9073E-12	0.25614	7.7052E-12
	RELION	Wilcoxon	285	0.6875	0.68713	1.5259E-02	0.02807	1.7607E-02
	EMAN2	Wilcoxon	285	0.6875	0.50705	5.3573E-48	0.98947	2.2960E-47
10345	CrYOLO	Wilcoxon	291	0.9027	0.03868	1.0302E-48	0.98282	6.1811E-48
	Topaz	Wilcoxon	291	0.9027	0.09484	1.4939E-47	0.8866	5.6021E-47
	Scipion	Wilcoxon	291	0.9027	0.85865	6.0934E-02	0.32302	6.7704E-02
	RELION	Wilcoxon	291	0.9027	0.90623	4.0027E-03	-0.09622	5.0033E-03
	EMAN2	Wilcoxon	291	0.9027	0.70119	6.7193E-42	0.83849	2.0158E-41
11056	CrYOLO	Wilcoxon	299	0.8180	0.51864	8.8662E-51	1	2.6599E-49
	Topaz	Wilcoxon	299	0.8180	0.48439	1.5624E-49	0.9398	1.1718E-48
	Scipion	Wilcoxon	299	0.8180	0.73528	8.3986E-32	0.58528	1.6797E-31
	RELION	Wilcoxon	299	0.8180	0.81038	4.2033E-03	0.10368	5.0439E-03
	EMAN2	Wilcoxon	299	0.8180	0.57915	6.7055E-45	0.8194	2.2352E-44

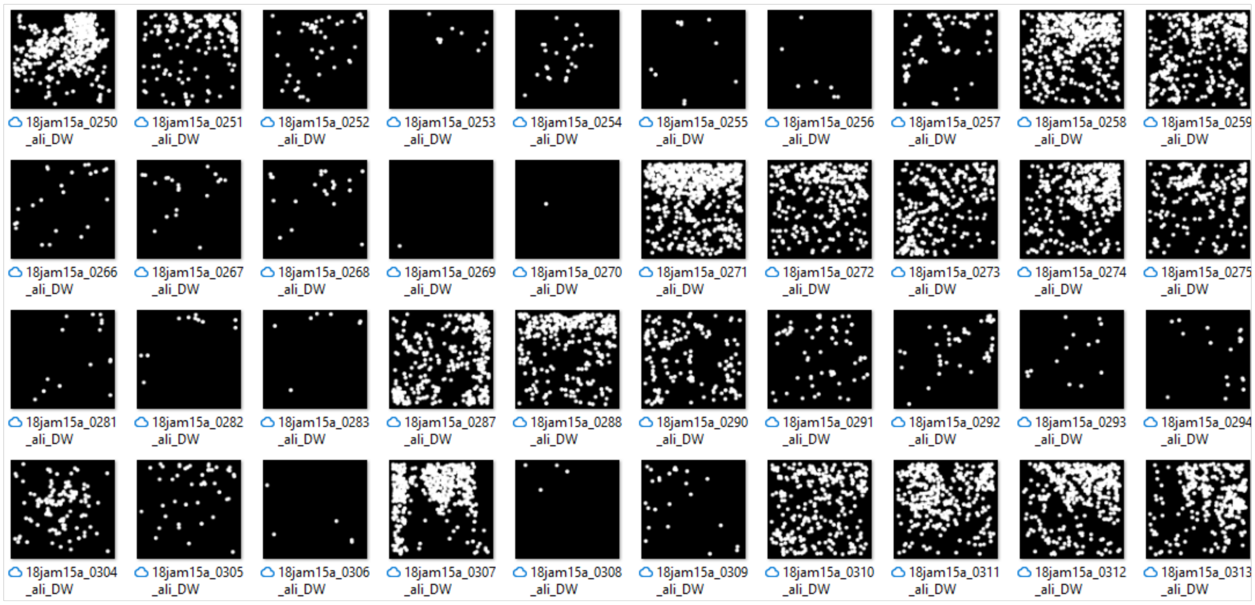

(a)

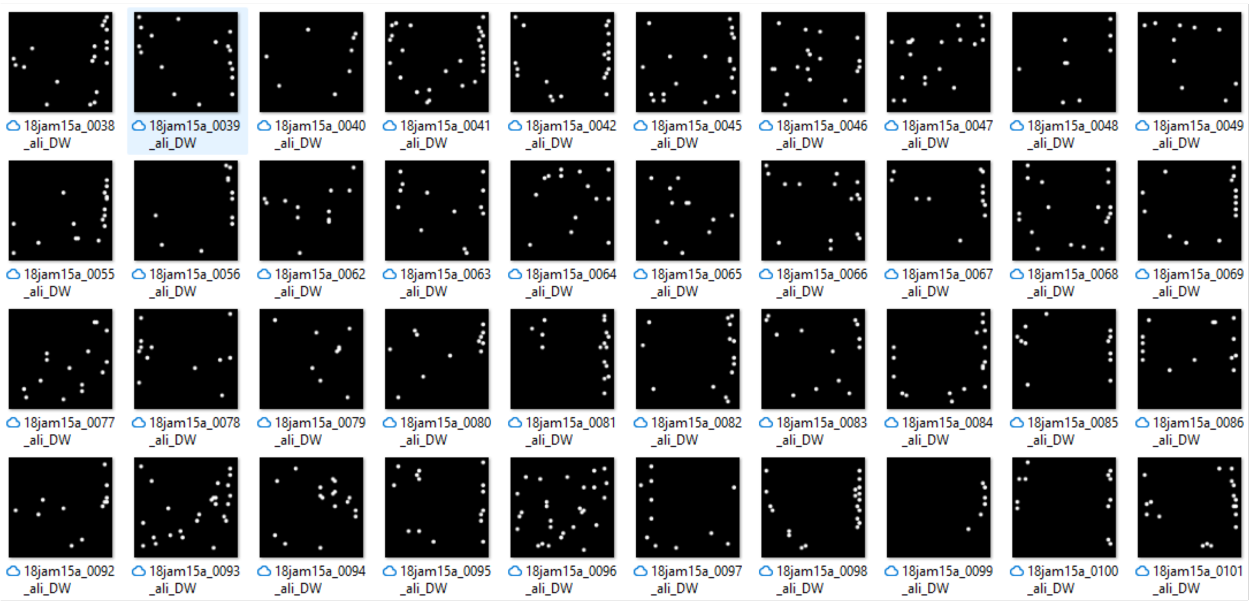

(b)

Supplementary Figure S1: Visualization of binary mask predictions for protein particles on EMPIAR-10345 micrographs, comparing (a) Topaz and (b) CrYOLO methods. (a) displays Topaz's output, where binary masks (white regions) highlight detected particles, revealing inconsistent performance: over-picking is evident in some micrographs with excessive mask overlaps, while under-picking occurs in others, missing true particles due to low sensitivity in noisy conditions. (b) presents CrYOLO's output, with a binary mask consistently showing under-picking across nearly all sample micrographs, underrepresenting the true number of protein particles, and leaving significant areas undetected. Both figures illustrate the limitations of these methods on the challenging EMPIAR-10345 dataset, characterized by low signal-to-noise ratios and structural heterogeneity.

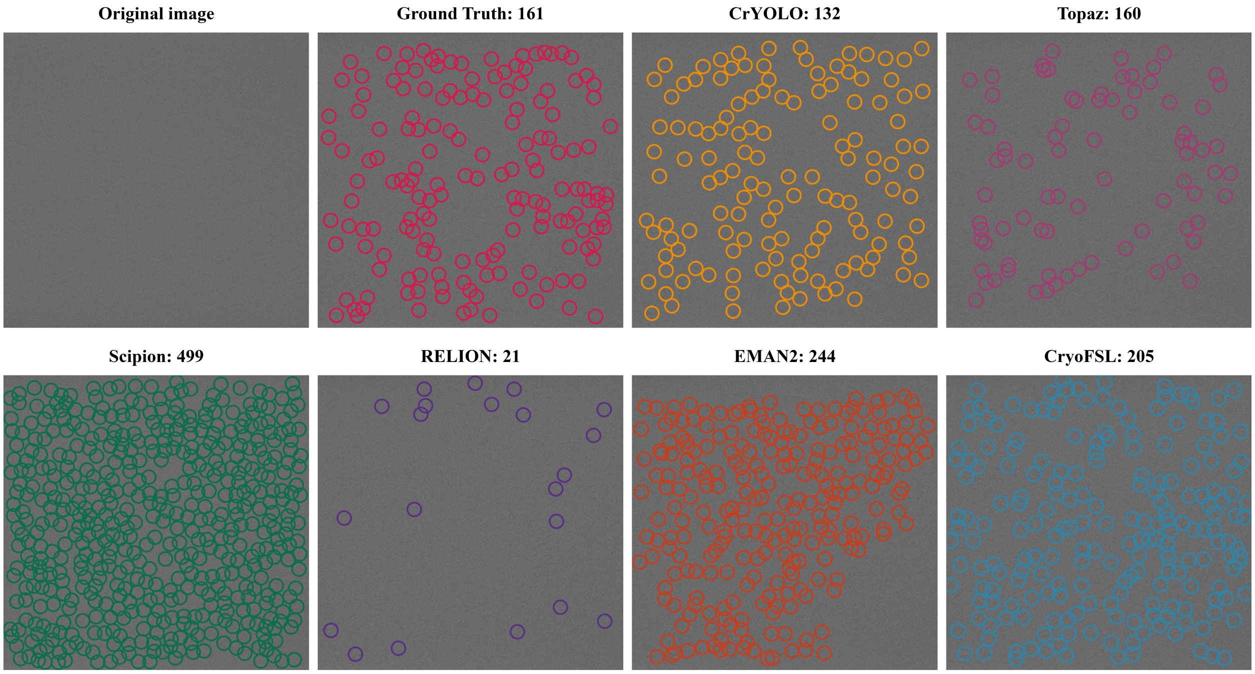

Supplementary Figure S2: Visualization of particle picking on EMPIAR-10093 using different methods with their particle counts.

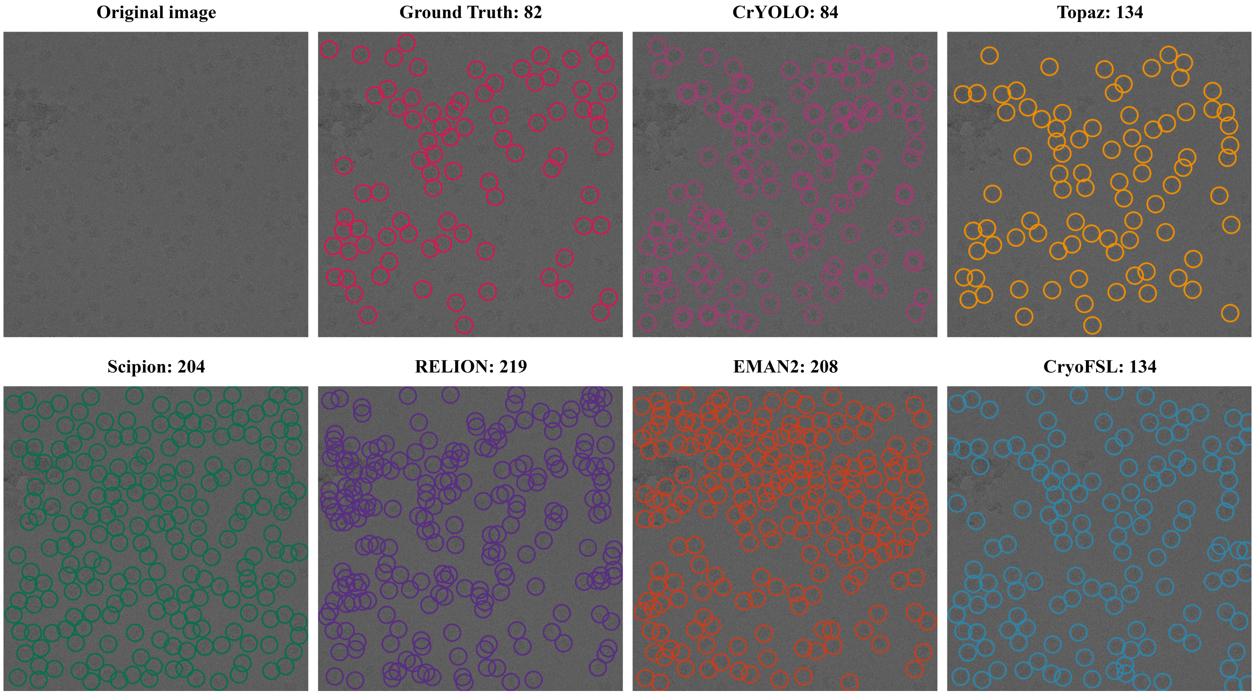

Supplementary Figure S3: Visualization of particle picking on EMPIAR-10028 using different methods with their particle counts.

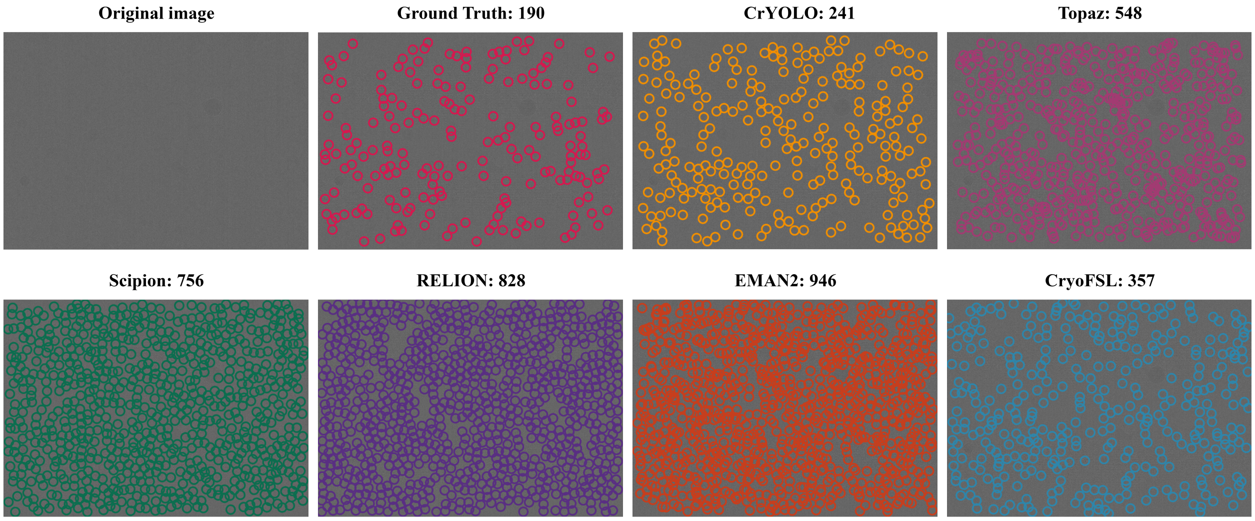

Supplementary Figure S4: Visualization of particle picking on EMPIAR-11056 using different methods with their particle counts.

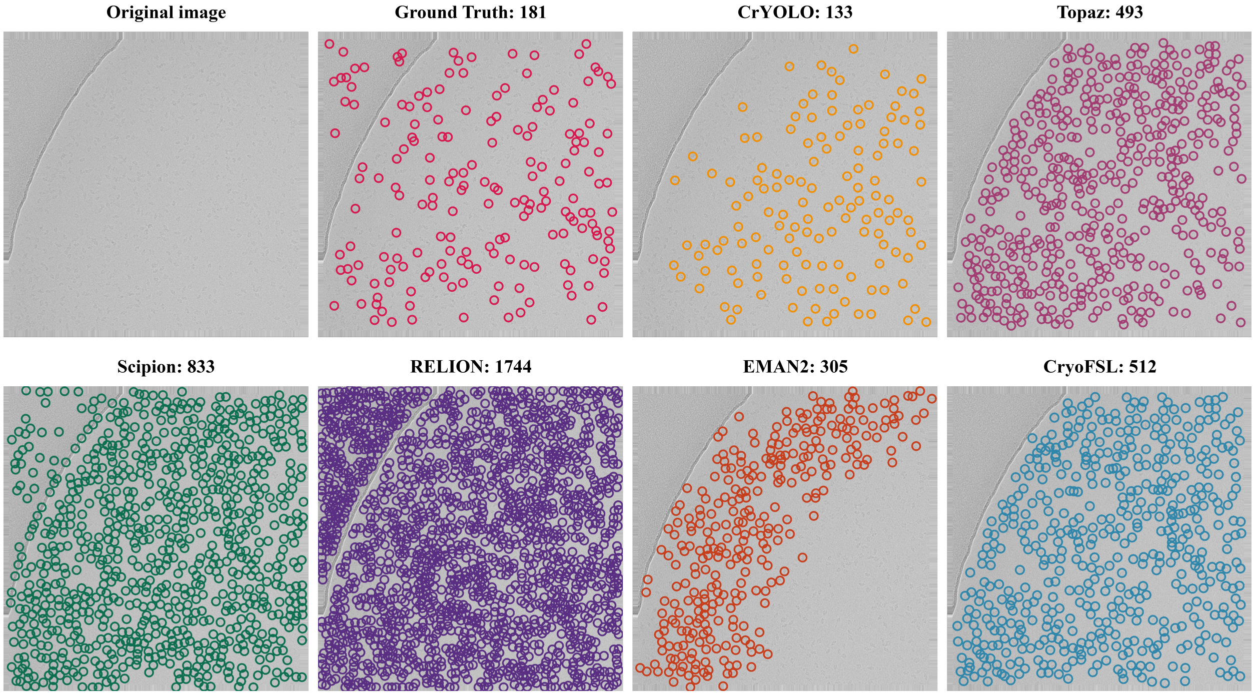

Supplementary Figure S5: Visualization of particle picking on EMPIAR-10017 using different methods with their particle counts.

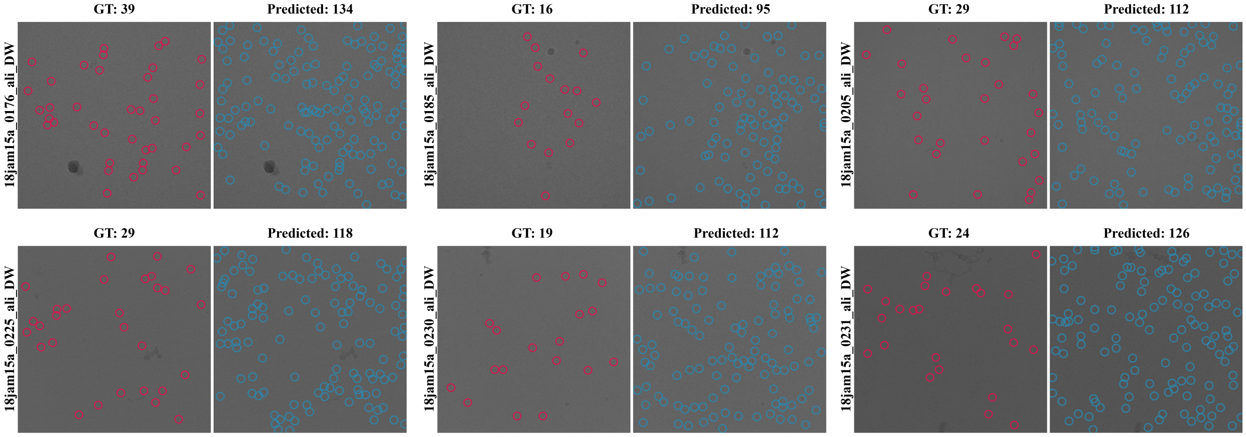

(a)

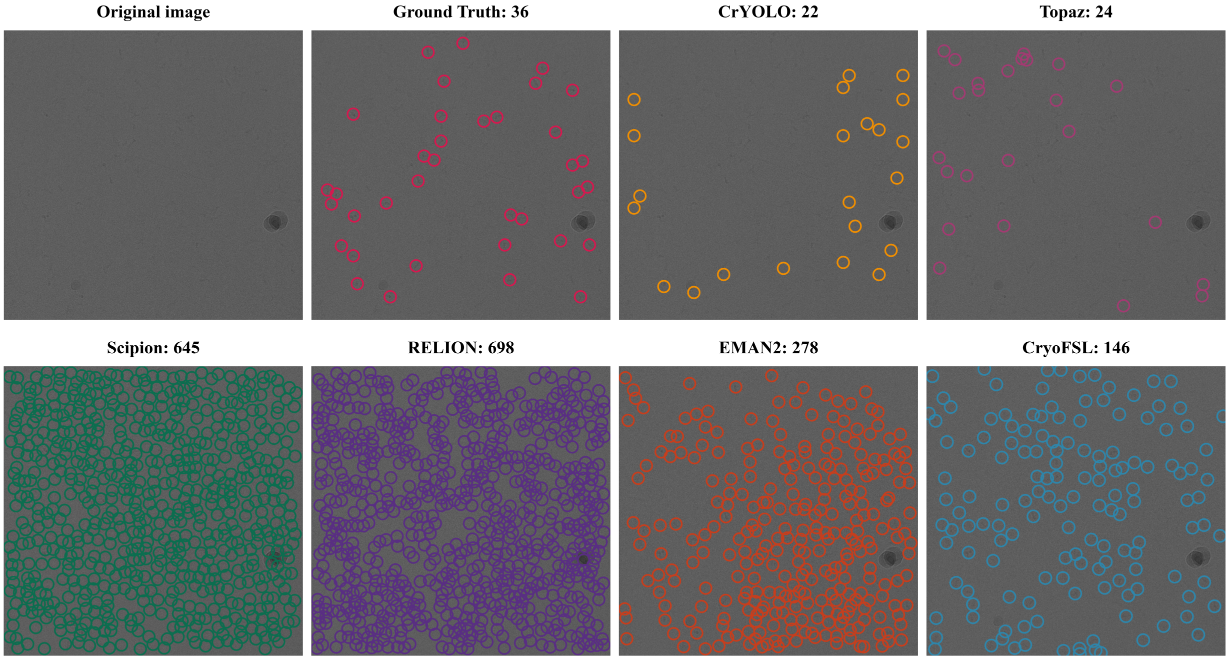

(b)

Supplementary Figure S6: Comparative analysis of particle detection across methods on EMPIAR-10345 dataset. (a) Displays multiple sample micrographs from the EMPIAR-10345 dataset, comparing GT annotations (left) with CryoFSL predictions (right), with particle numbers annotated alongside. CryoFSL accurately identifies GT particles and detects additional true positives (TPs) missed during GT curation, reflecting its high recall and robustness, though precision is lower due to these extra valid detections rather than false positives. (b) Detailed comparison of all six particle picking methods on sample micrograph 18jam15a_0309_ali_DW, illustrating distinct failure modes across approaches. CrYOLO and Topaz exhibit systematic under-picking, missing substantial numbers of true particles present in the ground truth. In contrast, template-based methods (Scipion, RELION, and EMAN2) demonstrate over-picking behavior, selecting numerous false positive regions that do not correspond to actual protein particles. The lower precision observed in these traditional methods stems from excessive false positive detections, fundamentally differing from CryoFSL's precision characteristics, which result from identifying legitimate particles missed during manual annotation rather than selecting non-particle regions. This comparison highlights the distinct nature of precision limitations across methods: CryoFSL's comprehensive true positive recovery versus traditional methods' false positive contamination.

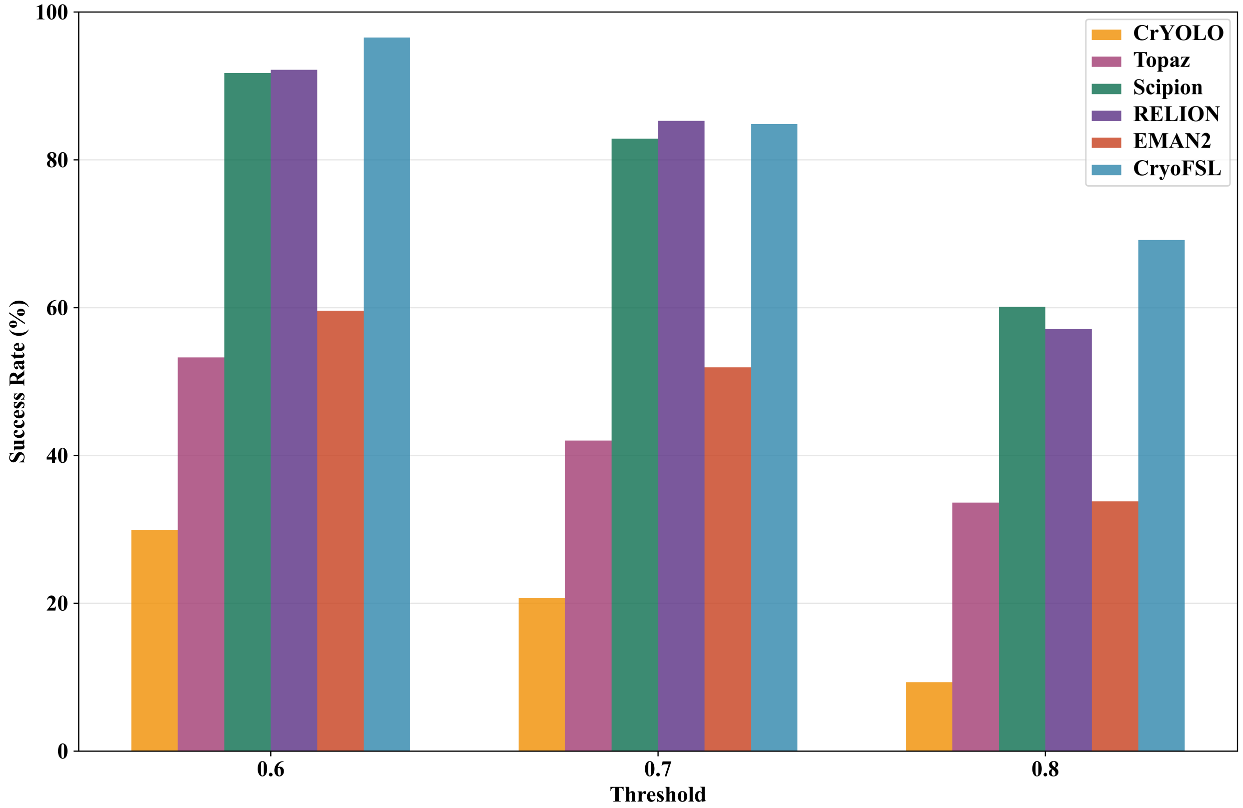

Supplementary Figure S7: Bar chart illustrating the recall success rate of different particle picking methods at thresholds 0.6, 0.7, and 0.8. Success rate is defined as the percentage of cases for which a method achieves a recall value equal to or exceeding predefined thresholds. The x-axis indicates the recall threshold values, and the y-axis represents the success rate in %. Each bar corresponds to a method's success rate at a specific threshold, with taller bars indicating a higher proportion of cases meeting or exceeding the recall criterion.

Threshold-based recall success rate evaluation

In order to determine stability under varying recall expectations, we computed the recall success rate, defined as the percentage of cases for which a method achieves a recall value equal to or exceeding predefined thresholds (0.6, 0.7, and 0.8). As shown in Supplementary Figure S7, CryoFSL outperforms all baselines across all thresholds, demonstrating not only high recall but also steady performance even under stringent conditions. At the moderate threshold of 0.6, CryoFSL achieved an outstanding 96.6% success rate with minimal variability (±3.9%), indicating near-universal capability to detect the majority of particles. This performance greatly surpasses deep learning methods like Topaz (53.3 ± 34.1%) and CrYOLO (29.9 ± 40.0%), while closely matching template-based methods such as RELION (92.2 ± 13.5%) and Scipion (91.7 ± 17.3%).

At a stringent threshold of 0.8, which represents a high confidence requirement for particle detection, CryoFSL sustains a 69.1% success rate, representing a 9.1% performance advantage over the next-best model (Scipion at 60.1%) and a staggering 7.4-fold improvement over CrYOLO (9.3%). The sharp decline in success rates for conventional methods as the recall threshold increases highlights their inconsistency in achieving recall. The steep variance (e.g., ±36.2% for Topaz and ±38.6% for EMAN2 at 0.8) further exposes the inconsistency in their results, with success highly dependent on the specific proteins or datasets. This clearly illustrates that our approach maintains reliable particle detection even under high-confidence requirements, setting it apart in terms of dependability and practical utility.

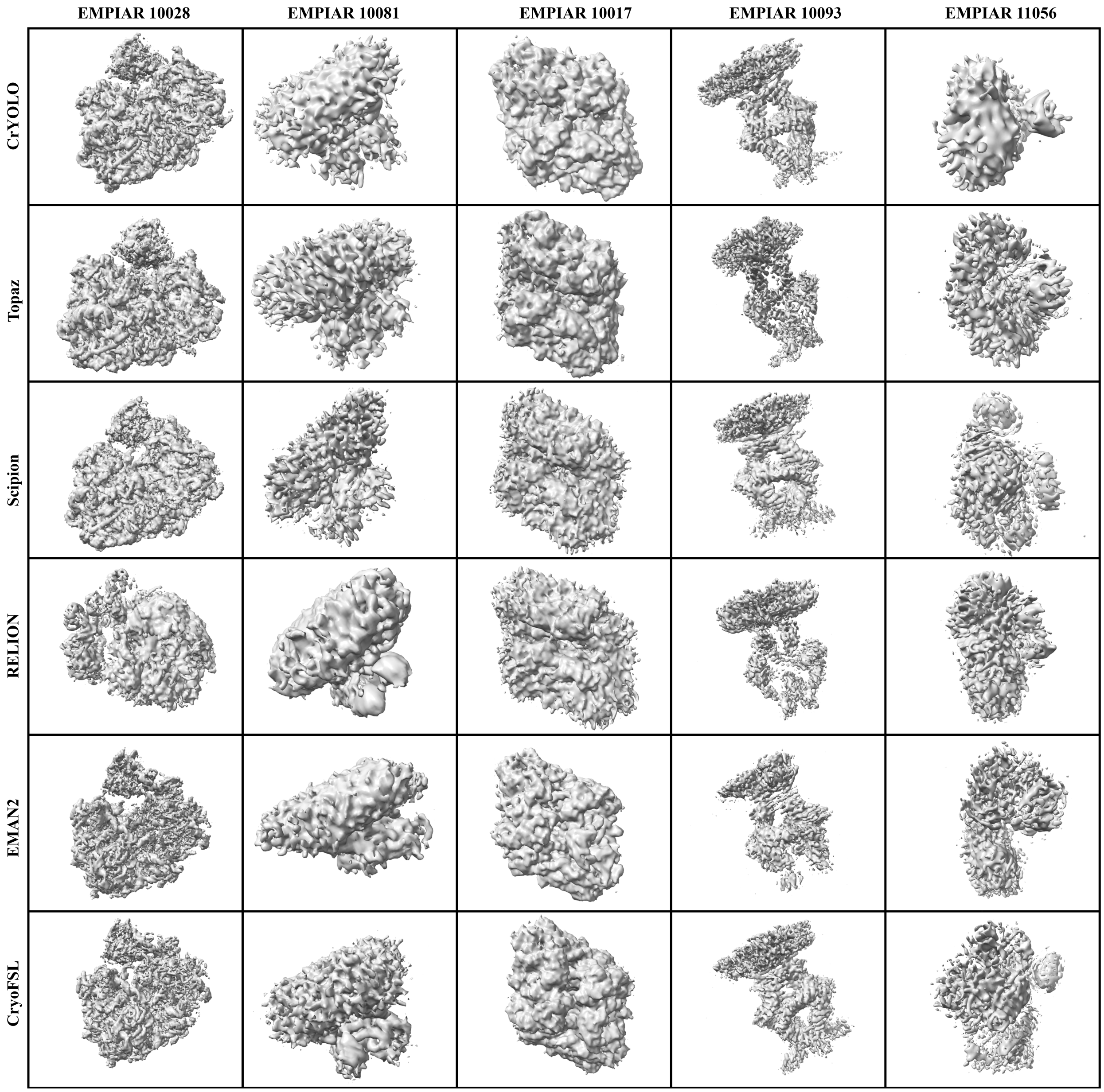

Supplementary Figure S8: Comparison results for density maps of particles picked by different methods across multiple EMPIAR IDs (10028, 10081, 10017, 10093, and 11056). CryoFSL outputs high-resolution density maps compared to other methods in most protein types.

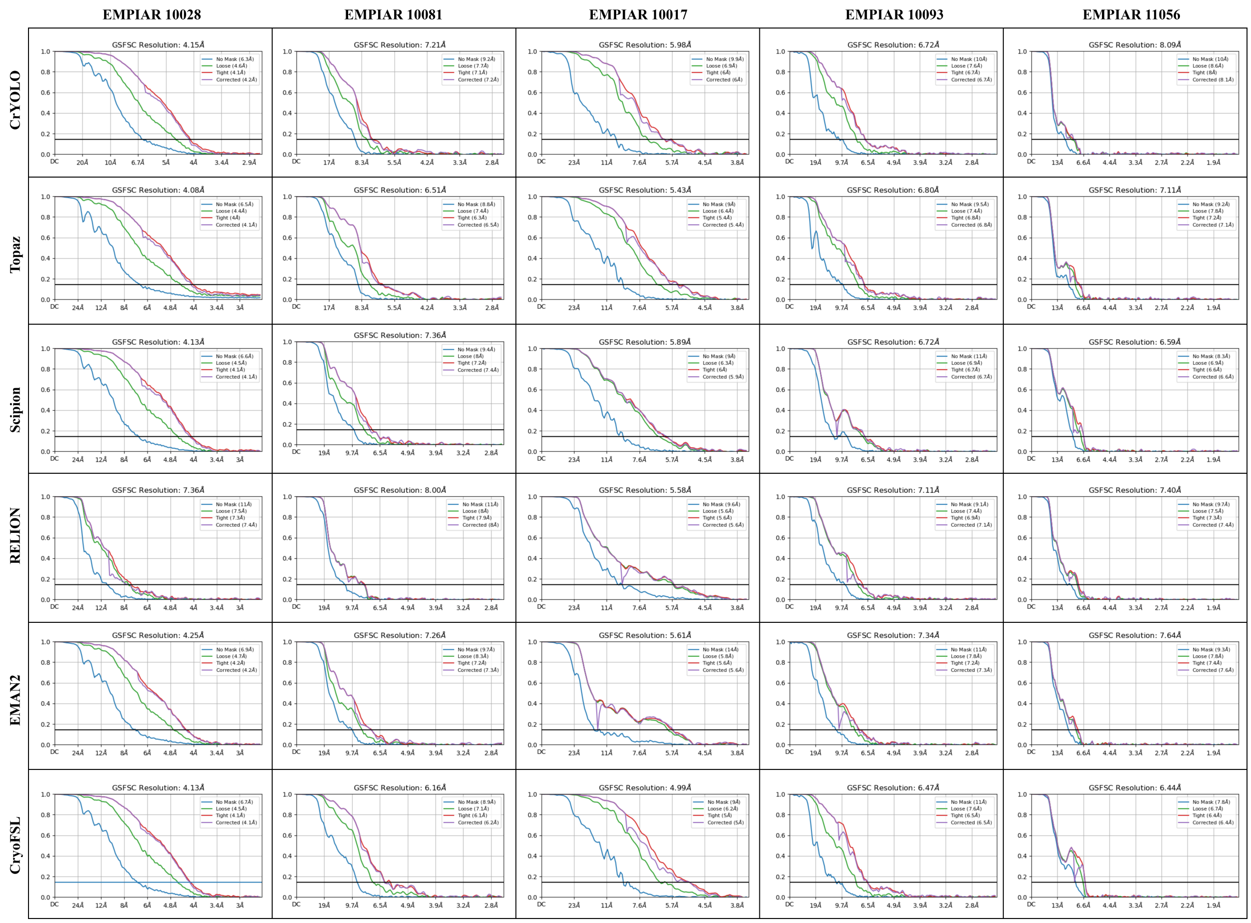

Supplementary Figure S9: Comparison results for resolution of density maps of particles picked by different methods across multiple EMPIAR IDs (10028, 10081, 10017, 10093, and 11056). CryoFSL has better resolution than other methods in most protein types.

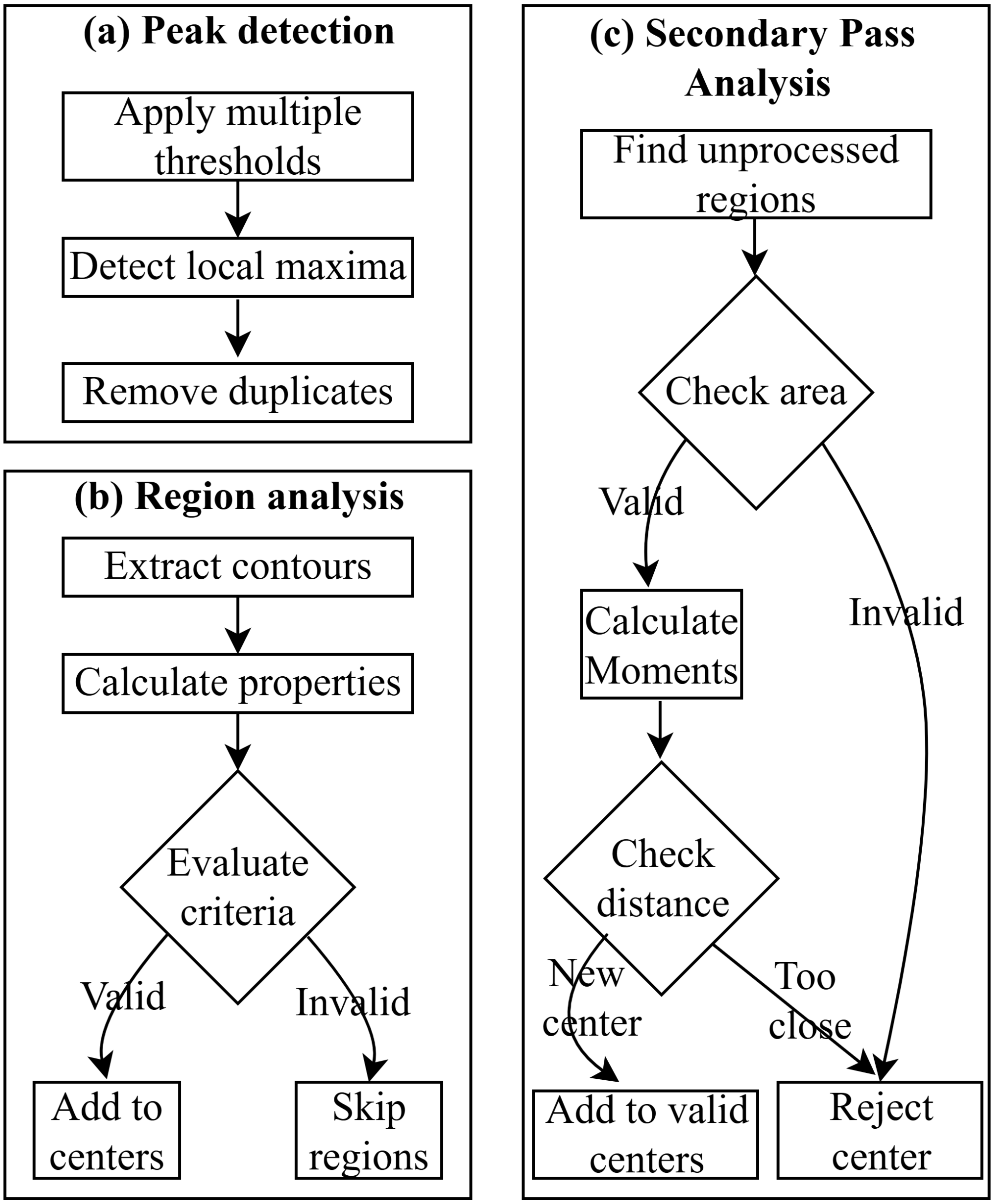

Supplementary Figure S10: Flowchart of the enhanced particle center generation algorithm for post-processing in CryoFSL. The pipeline consists of three main stages: (a) Peak detection module that applies multiple distance transform thresholds to detect local maxima and removes duplicate detections, (b) Region analysis module that extracts contours from watershed-segmented regions, calculates geometric properties, and validates regions based on area and circularity criteria, and (c) Secondary pass analysis module that identifies unprocessed regions and applies additional validation based on area and distance constraints to detect previously missed particles. Diamond shapes represent decision points with validation criteria, rectangular boxes indicate processing steps, and arrows show the sequential flow of operations. Valid regions are added to the final particle center list, while invalid regions are rejected or skipped. The complete algorithmic implementation with detailed parameters and mathematical formulations is provided in Supplementary Algorithm S1.

1 Supplementary Algorithm S1: Enhanced particle center generation

Input: Binary mask Image $B(x, y) \in (0, 1)$, expected particle radius r
Output: List of particle centers $\{(x_1, y_1), (x_2, y_2), \ldots, (x_n, y_n)\}$

1. Calculate the distance transform:
   $D(x, y) = \min\left\{\sqrt{(x - x')^2 + (y - y')^2} \mid B(x', y') = 0\right\}$
2. Multi-scale peak detection
   (a) Define thresholds $T_k = \alpha_k \cdot r$, where $\alpha_k \in \{0.1, 0.15, 0.2, 0.25\}$
   (b) For each T_k:
      i. Detect local maxima: $C_k = \{(x, y) \mid D(x, y) > T_k\}$
      ii. Store coordinates of peaks and remove duplicate coordinates: $C = \bigcup_k C_k$
3. Create markers $M(x, y)$ where each unique center in C is assigned a distinct label.
4. Perform watershed segmentation on $-D(x, y)$ using $M(x, y)$:
   $L(x, y) = i$, if (x, y) belongs to the ith region.
5. For each region R_i in $L(x, y)$:
   (a) Compute area A, perimeter P, and circularity Cir.
   (b) Validate regions based on $A \in [0.3\pi r^2, 2.2\pi r^2]$ and Cir > 0.5 or $A < 1.2\pi r^2$
   (c) For valid regions, compute centroids using moments:
   $$(c_x, c_y) = \left(\frac{M_{10}}{M_{00}}, \frac{M_{01}}{M_{00}}\right)$$
6. Create mask of unprocessed regions by subtracting processed regions from input mask
7. Detect new centers based on area and distance validation from existing centers
8. Returns the list of valid particle centers
